# Continuous colonization of the Atlantic coastal rain forests of South America from Amazônia

**DOI:** 10.1098/rspb.2024.1559

**Published:** 2025-01-22

**Authors:** James A. Nicholls, Jens J. Ringelberg, Kyle G. Dexter, Oriane Loiseau, Graham N. Stone, Phyllis D. Coley, Colin E. Hughes, Thomas A. Kursar, Erik J. M. Koenen, Flávia Garcia, Maristerra R. Lemes, Danilo R. M. Neves, María José Endara, Haroldo C. de Lima, Catherine A. Kidner, R. Toby Pennington

**Affiliations:** ^1^ Institute of Evolutionary Biology, University of Edinburgh, Edinburgh EH9 3FL, UK; ^2^ Royal Botanic Garden Edinburgh, Edinburgh EH3 5LR, UK; ^3^ Australian National Insect Collection, CSIRO, Canberra ACT 2601, Australia; ^4^ School of Geosciences, University of Edinburgh, Edinburgh EH9 3FF, UK; ^5^ Department of Systematic and Evolutionary Botany, University of Zurich, Zurich CH-8008, Switzerland; ^6^ Department of Life Sciences and Systems Biology, University of Turin, Torino 10124, Italy; ^7^ Department of Biology, University of Utah, Salt Lake City, UT 84112-0840, USA; ^8^ Departamento de Biologia Vegetal, Universidade Federal de Viçosa, Viçosa, MG 36570-900, Brazil; ^9^ Laboratório de Genética e Biologia Reprodutiva de Plantas,Coordenação de Biodiversidade, Instituto Nacional de Pesquisas da Amazonia, Manaus, AM 69067-375, Brazil; ^10^ Institute of Biological Sciences, Universidade Federal de Minas Gerais, Belo Horizonte 31270-901, Brazil; ^11^ Grupo de Investigación en Ecología y Evolución en los Trópicos- EETROP, Universidad de las Américas, Quito 170513, Ecuador; ^12^ Jardim Botânico do Rio de Janeiro, Rio de Janeiro 22460-030, Brazil; ^13^ Institute of Molecular Plant Sciences, University of Edinburgh, Edinburgh EH9 3BF, UK; ^14^ Department of Geography, University of Exeter, Exeter EX4 4QE, UK

**Keywords:** biogeography, Mata Atlântica, neotropics, *Inga*, dispersal

## Abstract

The two main extensions of rain forest in South America are the Amazon (Amazônia) and the Atlantic rain forest (Mata Atlântica), which are separated by a wide ‘dry diagonal’ of seasonal vegetation. We used the species-rich tree genus *Inga* to test if Amazônia—Mata Atlântica dispersals have been clustered during specific time periods corresponding to past, humid climates. We performed hybrid capture DNA sequencing of 810 nuclear loci for 453 accessions representing 164 species that included 62% of Mata Atlântica species and estimated a dated phylogeny for all accessions using maximum likelihood, and a species-level tree using coalescent methods. There have been 16–20 dispersal events to the Mata Atlântica from Amazônia with only one or two dispersals in the reverse direction. These events have occurred over the evolutionary history of *Inga*, with no evidence for temporal clustering, and model comparisons of alternative biogeographic histories and null simulations showing the timing of dispersal events matches a random expectation. Time-specific biogeographic corridors are not required to explain dispersal between Amazônia and the Mata Atlântica for rain forest trees such as *Inga*, which are likely to have used a dendritic net of gallery forests to cross the dry diagonal.

## 1. Introduction

The Atlantic coastal rain forests of Brazil (the ‘Mata Atlântica’) are highly species rich, estimated to contain *ca* 15 000 flowering plant species, of which 50% are endemic [1]. Here, using the species-rich genus *Inga* (Leguminosae) as an exemplar, we investigate the biogeographic processes that have led to this high Mata Atlântica species diversity.

**Figure 1.**
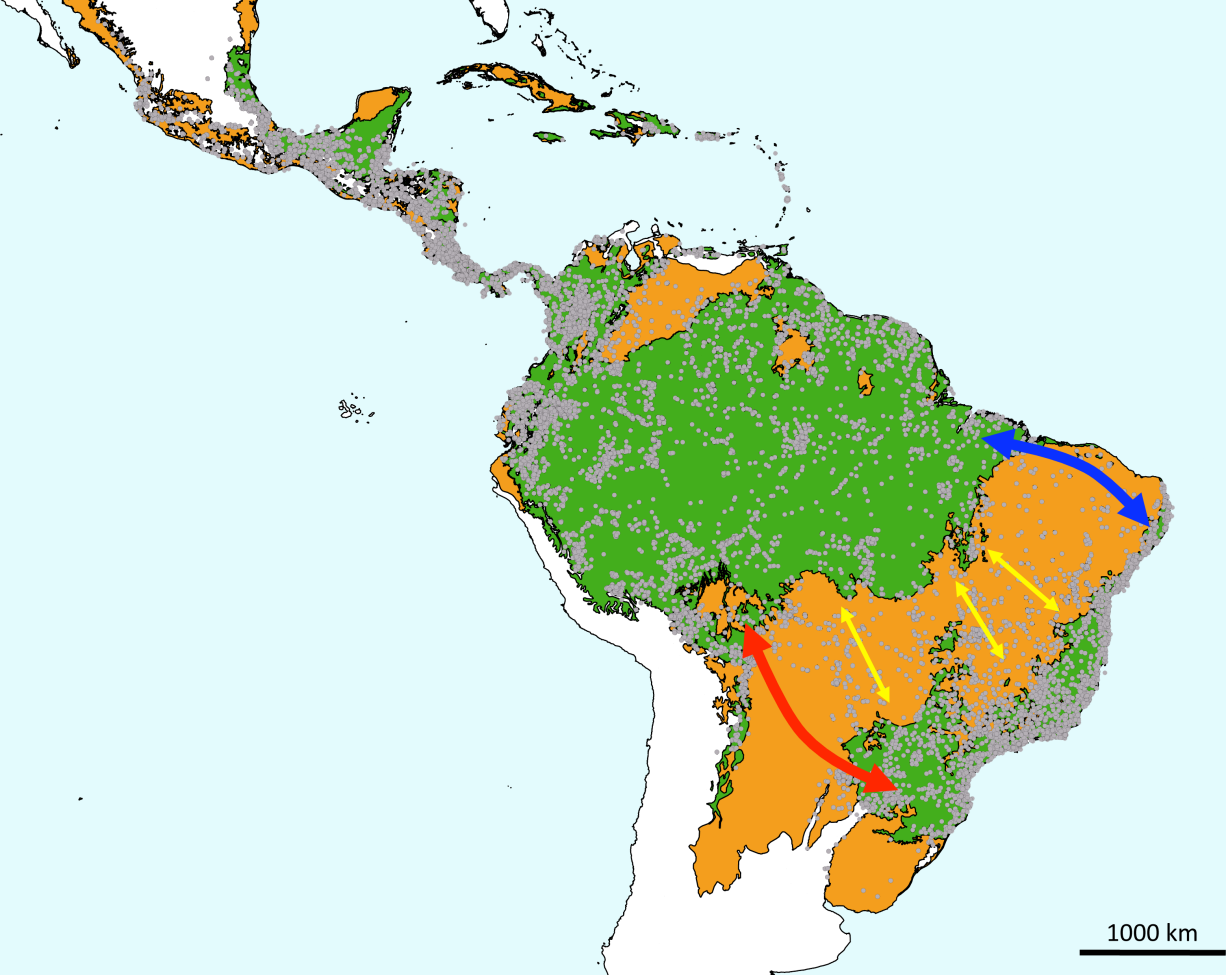
Map of major Latin American biomes showing the disjunct distribution of tropical wet forest and the intervening dry diagonal in South America, overlaid with *Inga* occurrence records (grey circles; based upon 39 312 validated records from the Global Biodiversity Information Facility). The map is adapted from the ecoregion map of Olson *et al*. [5] but with ecoregions reclassified into tropical rain forests and cloud forests (green) and dry biomes (savanna, dry forest, chaco woodlands, seasonally flooded grasslands; in orange). Classification of the savanna and dry forest biomes follows Pennington *et al*. [6]. Coloured arrows provide a schematic of proposed historical or ongoing connection routes between Amazônia and Mata Atlântica: blue = the NE route, red = the SE-NW route, yellow = the dendritic network of gallery forests traversing drier biomes; the thicker arrows of the NE and SE-NW routes reflects Ledo & Colli’s [7] summary of the importance of these routes.

Phytogeographic links between the Mata Atlântica and Amazônia, the two largest areas of rain forest in South America, have long been considered [2]. The Mata Atlântica and Amazônia are separated by an approximately 1000 km wide ‘dry diagonal’ comprising the woodlands of the chaco, the cerrado savannas and the caatinga dry forests of Brazil ([3,4]; see figure 1), which originated when South American climates started to dry in the early Miocene [8]. The origin of the high diversity and endemism of the Mata Atlântica has been attributed to its geographic isolation, complex topography and historical climate changes, which may have presented opportunities for diversification [2,9]. Some plant lineages found in both the Mata Atlântica and Amazônia have been inferred to have originated in the Mata Atlântica (e.g. *Leandra* Raddi [10]; *Vriesia* Hassk. [11,12]). In other cases, given that Amazônia has been regarded as ‘the primary source of Neotropical biodiversity’ [13], it is the most likely source area for many lineages that subsequently diversified in the Mata Atlântica (e.g. Protieae [14]).

The narrative of how individual species (e.g. [2]) or clades spanning the dry diagonal came to be distributed in both Amazônia and the Mata Atlântica has tended to be one emphasizing isolation of both areas with some specific, time-limited historical opportunities when they were connected. Some Amazônia—Mata Atlântica disjunctions have been explained by vicariance, with the early Miocene formation of the dry diagonal envisaged to have divided a hypothetically continuous, pre-existing rain forest (e.g. [15]). Subsequent to the formation of the dry diagonal, biogeographic discussion has focused on how this arid barrier may have been crossed by organisms that are confined to mesic areas. Links between Amazônia and the Mata Atlântica have largely been envisaged as occurring by mesic ‘connection routes’ that are generally considered to have been open during specific time windows in the past (e.g. [11,16,17]; see [7,9] for reviews).

The principal connection routes proposed are between the southern Mata Atlântica and western Amazônia (SE-NW route, traversing the southern cerrado and chaco), and between the northeastern Mata Atlântica and eastern Amazônia (NE route, traversing the caatinga and northern cerrado; [7,16]). These routes were thought to have been available when climate was more favourable for lowland, mesic biota in intervening areas of the dry diagonal. Batalha Filho *et al*. [16] suggested that the SE-NW route is older, occurring from the middle to late Miocene (i.e. *ca* 16−5 Mya), with the NE route more recent, in the Pliocene and Pleistocene (i.e. *ca* 5 Mya to present). A third possibility for dispersal is via the dendritic network of gallery forests that covers the dry diagonal [18], where rivers rising on the Brazilian shield drain both into the Amazon and the Mata Atlântica. This gallery forest network has been less emphasized in discussions of Amazônia—Mata Atlântica connections (but see [7,19,20]) and in a recent review is considered to be less significant than the SE-NW and NE alternatives (see Figure 1 in [7]). Although the temporal availability of the dry diagonal gallery forest network route has not been discussed, it seems likely that it has been available continuously, although the extent of these riverine forests may have been reduced during more arid historical periods, such as recent Pleistocene glaciations [18].

**Figure 2.**
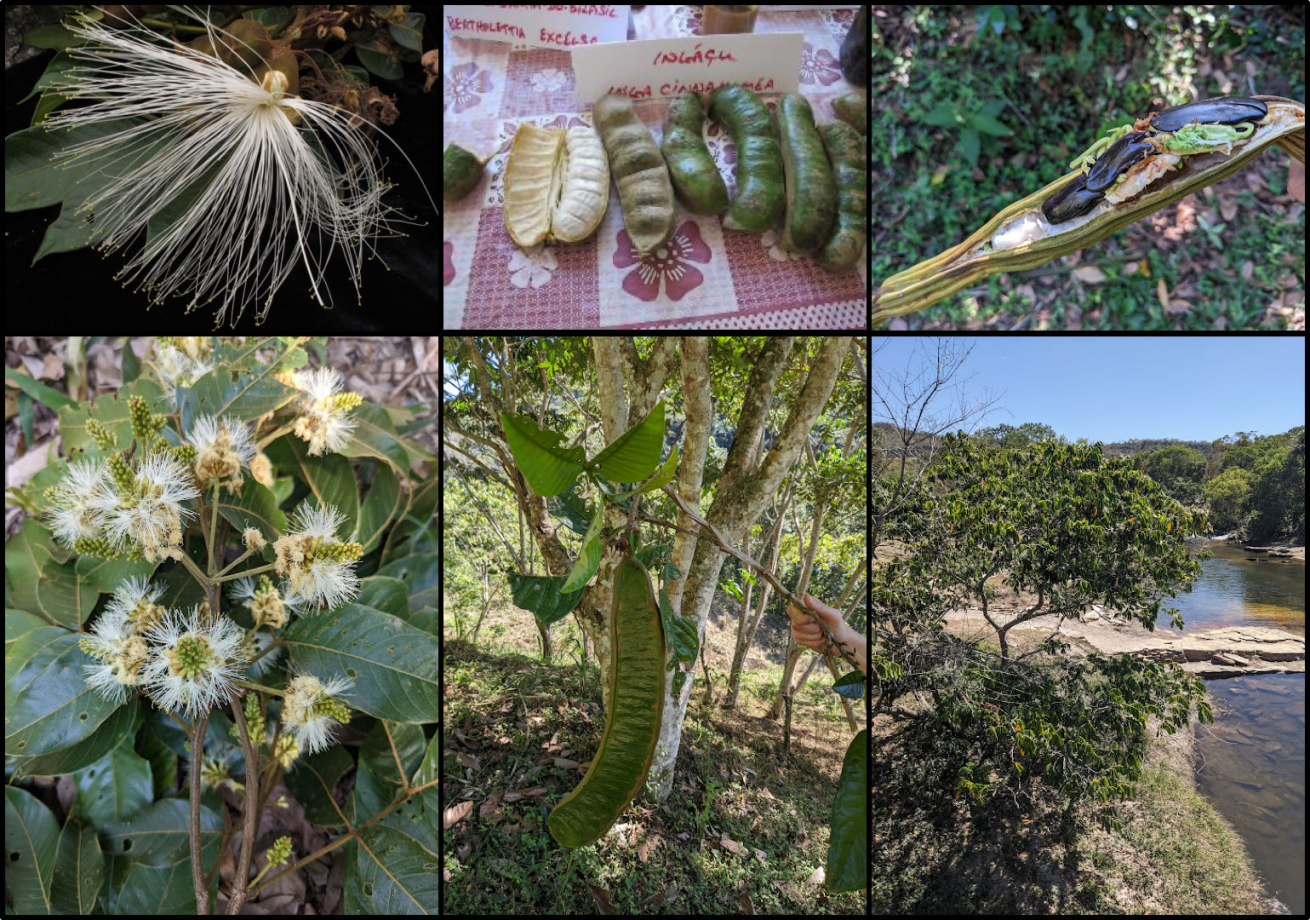
Images of *Inga* demonstrating biological characteristics and the humid forest adaptations of this genus. Clockwise from top left: flowers of *I. sessilis* from the Mata Atlântica; *I. cinnamomea* from Amazônia showing the fleshy sarcotesta, an adaptation facilitating primate dispersal; germinating naked *I. edulis* seeds, Amazônia, showing lack of drought adaptations; *I. affinis* growing alongside a river in Central Brazil, showing riverside habitat traversing drier cerrado vegetation; large edible legume of *I. spectabilis*, Amazônia; flowers of *I. lineata*, Amazônia. All photos by R.T. Pennington.

With *ca* 300 species in total distributed across the Neotropics (figure 1), *Inga* has significant numbers of species in Amazônia (including the Andean flanks of the Amazon; 145 species [21]) and in the Mata Atlântica (55 described or soon-to-be described species, of which 44 are endemic [21,22]). *Inga* species are characteristic of rain forest environments (see figure 2), with very few found in tropical dry biomes and none in the savanna or dry forest vegetation of the dry diagonal in central Brazil [23]. Distribution records of *Inga* species in central Brazil within the dry diagonal (figure 1) represent 21 species confined to gallery forests in the cerrado (but not endemic there; 10 are widespread in Amazônia, Mata Atlântica and central Brazil, eight in Amazônia and central Brazil, and three in Mata Atlântica and central Brazil [23]). These 21 species are not found in adjacent fire-prone savanna vegetation [23], as demonstrated by their occurrence records being clustered along rivers (electronic supplementary material, figure S1).

The fruits of *Inga* are indehiscent legumes that vary from 5 cm to 1 m long, but the fleshy-coated seeds are dispersed in all cases by primates or other mammals ([21]; figure 2). The restriction to humid environments may reflect that the fleshy, naked embryo that forms their seeds requires shade and high humidity for germination, and cannot survive desiccation ([21]; figure 2). This requirement of humidity for germination, coupled with a lack of dormancy, excludes *Inga* from seasonally dry environments and means that to move between Amazônia and the Mata Atlântica, *Inga* species must have dispersed within or between areas of rain forest.

Previous phylogenies of *Inga* were based upon Sanger sequencing of seven chloroplast loci and the nuclear ribosomal internal transcribed spacer region [24,25], did not include any accessions from the Mata Atlântica and were poorly resolved, likely because of the recent origin and rapid radiation of all extant *Inga* species. Studies that have dated the *Inga* radiation estimated a recent crown age of *ca* 10 million years (from 6 to 13 Mya [24,26,27]), post-dating the early Miocene establishment of the dry diagonal. These dates largely rule out vicariance explanations for the disjunctions within *Inga* between Amazônia and the Mata Atlântica but encompass a time window that potentially allows for dispersal between these rain forest areas along all three proposed connection routes. However, the lack of phylogenetic resolution possible with this small set of eight loci (including seven linked plastid loci) means that quantifying the timing, directionality and numbers of dispersal events between these two areas requires a much larger DNA sequence dataset than merely sequencing the same markers for Mata Atlântica species.

In this article, we generate a new, fully resolved, time-calibrated *Inga* phylogeny based on hybrid capture DNA sequencing of 810 nuclear loci, sampling 453 *Inga* accessions representing 164 species, including 62% of Mata Atlântica species. We use this phylogeny to investigate the regional biogeographic connections between the Mata Atlântica and Amazônia. Understanding and quantifying such regional biogeographical transitions requires dense taxon sampling, including multiple accessions within individual species that are widespread, coupled with a highly resolved phylogeny such as we generate here.

Using our *Inga* phylogeny, we examine the frequency and timing of dispersal events between the Mata Atlântica and Amazônia. Under a dispersal model along the older SE-NW route and given the age of the genus *Inga*, dispersal events are predicted to be clustered in time and occur only towards the base of the phylogeny. Under a NE dispersal route model, dispersal events are predicted to occur at shallower levels in the phylogeny, but also to be clustered in a discrete time-restricted period. In contrast, a model involving dispersal through the gallery forests of the dry diagonal would predict dispersal events to be more continuously distributed across the time frame encompassed by the *Inga* phylogeny.

## 2. Material and methods

### (a) Taxon sampling

We sampled 453 accessions (electronic supplementary material, table S1) that covered 164 species-level taxa of *Inga*, 55% of the estimated *ca* 300 species in the genus [21]. For the specific biogeographic questions addressed here, focusing on Amazônia and the Mata Atlântica, we sampled a good representation of the Amazon (*n* = 100 of 145 recognized species (69%), plus 27 morphospecies) and the Mata Atlântica (*n* = 32 of 52 recognized species (62%), plus two morphospecies). Our taxonomy primarily follows Pennington [21], with inclusion of some more recently described species (see [22,28–32]). However, in some instances the individual-level phylogenetic analyses suggested that Pennington [21] may have incorrectly synonymized some species/subspecies, so in those cases we treat them as distinct species (see electronic supplementary material, table S1). For the morphospecies, phylogenetic analyses at the individual level (see below) suggested that these taxa are independently evolving, evolutionarily significant units, which likely correspond to species. These could represent new species, but because in many cases our collections lack either flowers or fruit required for confident species delimitation, we prefer not to describe them formally or attempt to assign them existing species names given we have not covered all existing species in our phylogeny; for this study they are listed using field codes or codes indicating their affinity to named taxa. Of the 52 recognized Mata Atlântica species, 41 are endemic and we sampled 23 of these endemics. Of the 11 non-endemic species, all are also found in Amazônia and we sampled Mata Atlântica and Amazônian populations of nine of these. *Inga cayennensis* Sagot ex Benth. and *I. ingoides* (Rich) Willd. were only sampled in the Amazon. Furthermore, we sampled two of three known, but currently undescribed endemic Mata Atlântica morphospecies. We acknowledge that our species sampling is not complete, but we are confident it reflects the phylogenetic, morphological and geographic diversity of Amazônian and Mata Atlântica *Inga* and is sufficient to provide a minimum estimate of numbers of dispersal events amongst these areas, and an unbiased estimate of their timing (see §4).

### (b) Laboratory protocols

DNA extraction, library preparation and targeted enrichment followed the methods of Nicholls *et al*. ([33]; using the Qiagen DNeasy Plant Mini kit, Illumina TruSeq Nano DNA LT Sample Preparation kit, and MYcroarray MYBaits kit, respectively), with the exception that a larger bait set was used that additionally incorporated the bait set designed by Koenen *et al*. [34] for the wider mimosoid clade within which *Inga* is nested. Enriched libraries were split into seven different pools for sequencing: three pools of 24 samples each were sequenced on an Illumina MiSeq (v2 kit) with 250 bp paired-end reads, three pools of 96 samples each were run on an Illumina HiSeq (high output v4 kit) with 125 bp paired-end reads and one pool of 96 samples was run on an Illumina HiSeq 4000 with 150 bp paired-end reads.

### (c) Bioinformatics

Analysis of the captured reads and generation of consensus sequences for each locus for each sample followed the same mapping methods of Nicholls *et al*. [33]. The alignment score threshold for bowtie2 [35] was optimized for data derived from each type of sequencing run, using 320 for the three MiSeq runs, 130 for the three HiSeq high output runs and 170 for the HiSeq 4000 run. The full bait set comprises 1320 loci. Of these, 820 baits were confirmed to show reliable phylogenetic signal by testing for the placement of *Zygia* as an outgroup, as in Nicholls *et al*. [33]. Of this set, 810 had sufficient sequence data across accessions to be used for analysis here. The raw reads for each sample have been deposited at NCBI’s Short Read Archive (https://www.ncbi.nlm.nih.gov/bioproject/) under the BioProject codes PRJEB8722 and PRJNA683762 (individual accessions listed in electronic supplementary material, table S1).

### (d) Phylogeny estimation and dating

We used three phylogenetic trees in our analyses: (i) a tree sampling all individual accessions, which is important to visualize dispersal events that have occurred within species, (ii) a species tree sampling one individual per species constructed using coalescent methods (ASTRAL) and (iii) a species tree built using a concatenation approach in BEAST, which provided dates for temporal calibration of the individual-level and species-level ASTRAL trees. Given that the ASTRAL and BEAST species trees differed slightly in topology, we conducted biogeographic analyses on both, to assess the consistency of our results.

#### (i) Phylogenetic inference

We used the maximum likelihood implemented in the software IQ-TREE 2 [36] to infer the individual-level phylogenetic relationships of the 454 accessions from our dataset of 810 genes. This individual-level tree allows inference of exchanges between Amazônia and the Mata Atlântica that occur within species that are widespread in both areas. We performed a partitioned analysis [37] after inferring the best-partition scheme for the 810 genes and the best substitution model for each partition using the ModelFinder module implemented in IQ-TREE 2 [38]. We performed 1000 bootstrap replicates with the ultrafast bootstrap approximation [39].

*Inga* represents a recent species radiation, which may lead to incomplete lineage sorting in the loci we have used for phylogenetic reconstruction. For this reason, we also inferred a species-level phylogeny with ASTRAL-II [40] after gene trees (each containing all accessions) were estimated for each of the 810 genes in IQ-TREE 2, using the ModelFinder module to estimate the best substitution model for each locus. Because ASTRAL branches do not represent proper branch lengths (i.e. substitutions), but instead coalescent units, they cannot be used to produce a dated phylogeny. We therefore used this ASTRAL species tree as a topological constraint for a new maximum likelihood phylogenetic inference in IQ-TREE 2 that estimated branch lengths for subsequent temporal calibration. For this IQ-TREE analysis, we pruned the alignment to retain only a single individual per species (samples confidently identified as their respective species and with high-quality sequence data were selected; see electronic supplementary material, table S1) and the same parameters as the first ML analysis were used, except that the topology was fixed and only branch lengths were estimated. We focus on this coalescent species-level phylogeny, for example in our BioGeoBEARS analyses, because we consider it more likely to be accurate in the face of incomplete lineage sorting within *Inga*’s recent radiation. In addition, we report analyses based on a BEAST [41] phylogeny that was used for divergence time estimation; this provided a test of robustness because its topology differed slightly from the ASTRAL species tree (see §2dii).

#### (ii) Divergence time estimation

**Figure 3.**
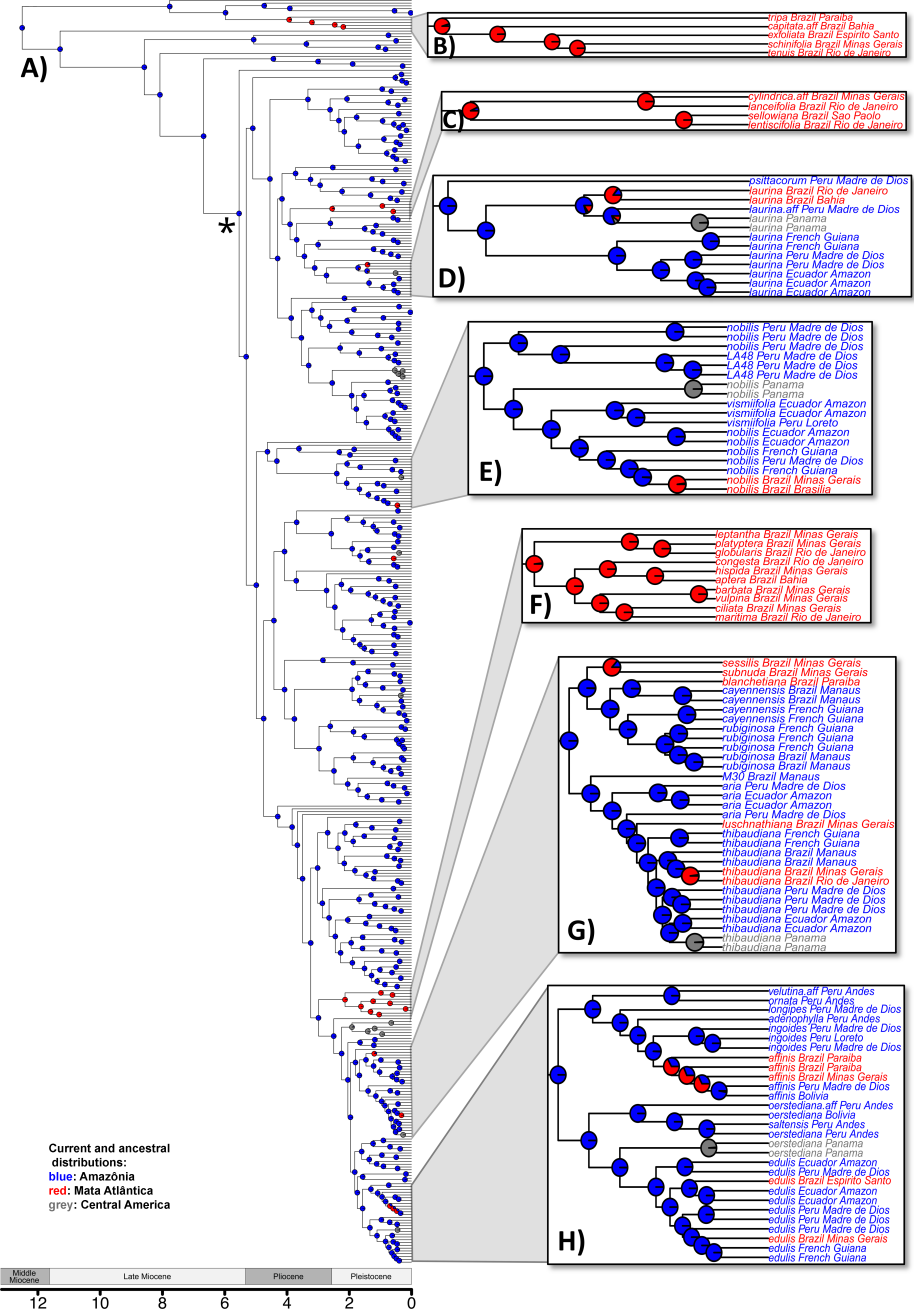
Maximum likelihood ancestral state reconstruction of biogeographic history on an *Inga* phylogeny that includes 453 accessions. Inserts (B*-*H) highlight selected clades that are relevant to points discussed in the text. Pie charts at internal nodes are coloured by the probability of different ancestral states, and tip labels within inserts are coloured by where the individual occurred: blue = Amazônia; red = Mata Atlântica; grey = Central America. The asterisk indicates the clade with a faster clock rate. Scale bar is in millions of years, alongside geological epochs. See electronic supplementary material, figure S2 for a version of this phylogeny with information for all tip labels.

We time-calibrated the individual-level and the ASTRAL-constrained IQ-TREE species-level phylogenetic trees using penalized likelihood implemented in the program treePL [42], employing cross-validation to estimate the best value of the smoothing parameter. In both analyses we constrained the crown node of *Inga* to an age of 12.67 Ma and the root node to an age of 17.55 Ma (the split between *Inga* and *Zygia s.s*.), following the results of a prior BEAST analysis of the species-level dataset [27] that used a fixed local clock model and two separate partitions to account for the faster rate of evolution in the majority of the genus (the clade highlighted by the asterisk in figure 3) compared with the earliest-diverging lineages. These crown and root node age calibrations were derived from the results of a molecular dating analysis of the higher-level clade that contains *Inga* (i.e. legume subfamily Caesalpinioideae) using seven fossil calibrations [27].

### (e) Reconstructing dispersal history and testing for time-clustered dispersal

#### (i) Individual-level phylogeny

We used hidden Markov models in corHMM [43] to reconstruct ancestral states on the individual-level phylogeny because this enables us to visualize several transitions between Amazônia and Mata Atlântica that occur within single species that are widespread in both areas. Using species-level phylogenies may miss some dispersal events and mis-infer their directionality. For analyses at the individual level, all individuals were assigned a state, either Mata Atlântica (*n* = 41) or not (*n* = 412), based on where they had been collected. Nearly all non-Mata Atlântica individuals were collected in Amazônia (*n* = 380), with relatively few individuals coming from Central America (*n* = 32). Because of our sparser sampling in Central America and our focus on dispersal between Amazônia and the Mata Atlântica, in the following analyses we excluded Central American species. Additional analyses including Central American accessions did not change our understanding of dispersal between Amazônia and the Mata Atlântica, because colonizations of Central America from Amazônia appear to be evolutionarily independent of colonizations of the Mata Atlântica in all cases (see electronic supplementary material, figure S2).

We tested whether equal rates (ER) or all rates different (ARD) models of character state change fitted our data better using the Akaike Information Criterion, corrected for small sample size (AICc; see electronic supplementary material, table S2). The ER model performed best and so was used for reconstructing ancestral states. We did not allow for internal nodes to be polymorphic, i.e. to be modelled as occurring in both Amazônia and Mata Atlântica, because this is not appropriate for terminals that represent geographically restricted populations. We then used stochastic character mapping (using corHMM’s makeSimmap function) to simulate 100 character evolution histories, and obtained the total number and ages of dispersal events for each simulation.

For each of the 100 stochastic character mappings of dispersal history, we then used a randomization-based approach to test for time-limited dispersal with the individual-level phylogeny. If dispersal between the two forests depended on time-limited connections between them, we would expect dispersal events to be clustered in time. We determined a null expectation for timing of dispersals by randomly placing the same number of dispersal events on the phylogeny as observed in a given stochastic character mapping. Our null model assumes a constant probability of dispersal as a function of time, and therefore that the probability of a dispersal event falling on a given branch in the phylogeny depends on its length. The timing of the dispersal events on a given branch was then determined by drawing from uniform distributions with minimum and maximum values that corresponded to the age of the parent and child node of the branch. The expected null distribution of dispersal event times was generated 10 000 times (cf. [44]). We used these to calculate the null expectations for the median and variance of the ages of dispersal events for each stochastic character mapping. We also counted the number of dispersal events in each randomization that fell within 1-million-year time bins. We then compared the summary statistics and number of dispersal events to those from the null expectation using two-tailed tests, whereby the observed value of a given statistic was considered to be significantly non-random if it fell outside the 95% quantiles of the values from the 10 000 randomizations. These statistical tests were repeated 100 times.

#### (ii) Species-level phylogeny

We reconstructed dispersal history on the species-level phylogeny, investigated numbers of Amazônia—Mata Atlântica dispersal events and tested for their time-stratified distribution using BioGeoBEARS [45–47]. These analyses were done on both the ASTRAL-generated coalescent and BEAST species-level phylogenies. We did not implement this approach on the individual-level tree because BioGeoBEARS is not specifically designed for phylogenies in which tips represent individuals rather than species [45]. After first selecting the best-fitting of the six standard non-time-stratified models in BioGeoBEARS (results in electronic supplementary material, table S4), we tested a series of models designed to capture scenarios of time-stratified and non-time-stratified dispersal based on the predictions of timings of the SE-NW and NE routes (outlined in electronic supplementary material, table S3). Following Dupin *et al*. [47], time-stratified dispersal was specified using time period-specific dispersal multiplier matrices (electronic supplementary material, table S3), and model fit was assessed using AICc scores and the behaviour of the free parameter w. We tested three different time stratifications: two equal time periods (0−6.33 and 6.33−12.67 Ma), three equal time periods (0−4.22, 4.22−8.44 and 8.44−12.67 Ma) and two time periods with a cut-off of 5 Ma (the approximate date when the SE-NW route is thought to have closed and the NE route to have opened; see §1). These are designed to capture if older dispersal (SE-NW route) was more frequent or if recent dispersal (NE route) was more frequent, with the three-period model additionally testing if both SE-NW and NE routes show peaks of migration with a period of lesser migration in-between when neither route may have been available. Internal nodes were allowed to be polymorphic (i.e. to occur in Amazônia and the Mata Atlântica). To infer the total number of dispersal events, the best-fitting model was used for Biogeographical Stochastic Mapping with 100 simulations [47], as well as to visualize the probabilities of the three states (Amazônia, Mata Atlântica or both) across all nodes in the phylogeny.

## 3. Results

### (a) *Inga* phylogeny

**Figure 4.**
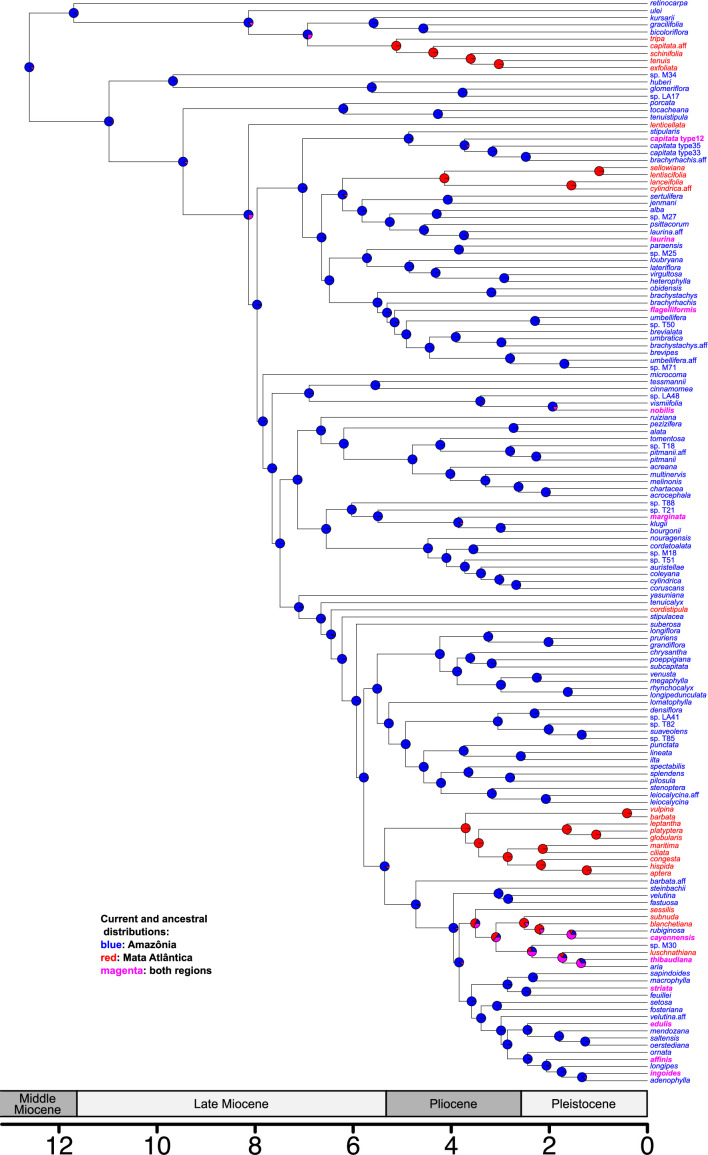
Maximum likelihood ancestral state reconstruction of biogeographic history on a species-level phylogeny of *Inga*. Tips in the phylogeny are coloured based on where the species occurs: blue = Amazônia; red = Mata Atlântica; bolded magenta species occur in both Amazônia and the Mata Atlântica. Pie charts at internal nodes are coloured by the probability of different ancestral states. Scale bar is in millions of years, alongside geological epochs. Central American species are excluded here for clarity but included in electronic supplementary material, figure S3.

We used IQ-TREE 2 [36] to infer an individual-level maximum likelihood phylogeny for the 453 sampled *Inga* accessions (figure 3, electronic supplementary material, figure S2) and a coalescent approach to develop a species-level phylogenetic tree (figure 4; electronic supplementary material, figure S3). The majority of nodes in both the individual-level phylogeny (91%) and species-level phylogeny (60%) had greater than 95% bootstrap support, with a further 5%/12% of nodes (individual/species tree, respectively) having between 85 and 95% support, and 2%/12% with 70–85% support (see electronic supplementary material, figures S2 and S3). Importantly for subsequent analyses, the more weakly supported nodes were not associated with branches upon which dispersal events between the Amazon and Mata Atlântica forests were modelled to occur. The coalescent species-level phylogeny shows incongruence when compared with the BEAST species-level phylogeny in the placement of species in two main *Inga* subclades (electronic supplementary material, figure S4). We focus on the coalescent species-level phylogeny (figure 4) here, for example in our BioGeoBEARS analyses, because we consider it more likely to be accurate in the face of incomplete lineage sorting, which is likely in *Inga*’s recent radiation, but we also report analyses based on the BEAST phylogeny.

### (b) Reconstructing dispersal history and testing for time-clustered dispersal

#### (i) Individual-level phylogeny

In the individual-level phylogeny (figure 3*a*), there was an average of 19.7 independent dispersals from Amazônia to the Mata Atlântica across the 100 simulated character histories and 2.2 dispersals in the opposite direction (one of which is always found within *Inga affinis* Steud.; figure 3*h*; electronic supplementary material, table S5). For 9 of the 11 species that occur in both Amazônia and the Mata Atlântica, multiple individuals were sampled spanning both areas, and in all but one case (*I. affinis*; figure 3*h*), the most recent common ancestor (MRCA) of the species is reconstructed as occurring in Amazônia. Of the eight species with a reconstructed Amazônian origin, five have multiple individuals sampled in the Mata Atlântica (*I. laurina* (Sw.) Willd., figure 3*d*; *I. nobilis* Willd., figure 3*e*; *I. thibaudiana* DC., figure 3*g*; *I. marginata* Willd., not highlighted and *I. edulis* Mart., figure 3*h*). In four of those cases, the multiple individuals sampled in the Mata Atlântica are each other’s closest relatives (i.e. are monophyletic) implying a single dispersal. In one species, *I. edulis*, there appear to have been two independent dispersals to the Mata Atlântica (figure 3*h*). The remaining dispersal events relate to species that occur in only one of the forests and gave rise to 25 species that only occur in the Mata Atlântica. In four cases, these dispersal events have been followed by speciation, giving rise to ‘Mata Atlântica clades’ comprising two (figure 3*g*), four (figure 3*c*), five (figure 3*b*) and ten species (figure 3*f*), respectively.

**Figure 5.**
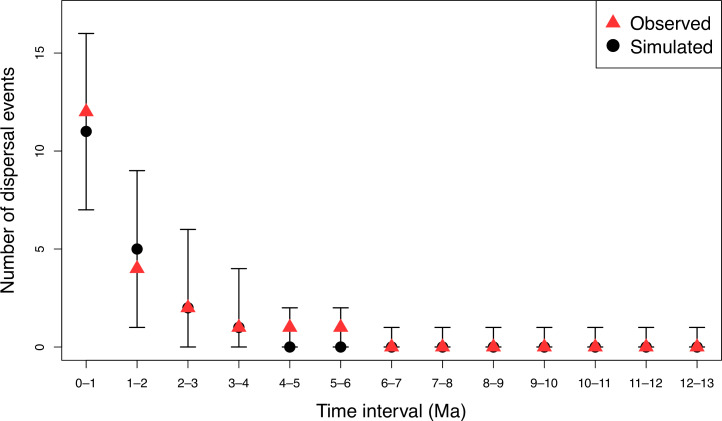
Observed number of dispersal events within 1-million-year time periods (red triangles) versus null expectation (median = black circles, error bars indicate 95% confidence intervals) based on 10 000 simulations of randomly placing dispersal events across the individual-level *Inga* phylogeny.

The observed dispersal events between the Amazon and the Mata Atlântica occur over a range of timescales (0.025–0.975 quantile of ages of dispersal events across 100 stochastic mappings: 0.01–5.62 Ma), and they appear to be clustered recently in time (median age = 0.79 Ma) (red triangles, figure 5). However, this observed distribution of dispersal events is similar to what would be expected if dispersals were to be scattered randomly along the branches on the phylogeny (black circles, figure 5).

The median of the observed distribution of ages of dispersal events was not significantly different from null expectations in all 100 stochastic mappings, and the variance was not different from null expectations in 95 of the 100 stochastic mappings. Furthermore, no 1-million-year time bin contained more or fewer dispersal events than expected at random (see figure 5 for observed and expected counts of dispersal events within 1-million-year time bins, comparing the median across the 100 stochastic character maps versus the medians from 100 sets of 10 000 simulations). Thus, our stochastic simulations strongly suggest that the distribution of dispersal events over the evolutionary history of *Inga* is statistically indistinguishable from the null expectation of no temporal variation in the probability of dispersal between the two forest regions.

#### (ii) Species-level phylogeny

Our model testing in BioGeoBEARS suggests that a single rate model of dispersal fits our data best and that there is no evidence of dispersal events clustered through time based on our species-level coalescent and BEAST phylogenies. DEC+j is the best model and performs better than any time-stratified model (electronic supplementary material, table S4). Whilst positive values of the free w parameter suggest there is limited support for some time-stratified models [47], these are not always for the same model (i.e. with one tree there is more support for higher dispersal in the oldest time period, with the other tree the reverse; electronic supplementary material, table S4). Overall, based on AIC scores there is not a single time-stratified model that outperforms the standard DEC+j model.

The pattern and number of dispersal events found on the individual-level phylogeny were mirrored in our BioGeoBEARS analyses of species-level phylogenies. The mean number of inferred dispersal events from Amazônia to Mata Atlântica varied from 16.3 (BEAST species tree) to 16.0 (coalescent species tree) with a mean of 1.3–2.2 inferred dispersal events from the Mata Atlântica to Amazônia (electronic supplementary material, table S5).

## 4. Discussion

We set out to discover if dispersal events in *Inga* between the Mata Atlântica and Amazônia are clustered in time, matching proposed SE-NW and NE connection routes, or are not time limited and therefore more consistent with a gallery forest route via the dry diagonal. Our analyses, using stochastic character mapping and null model randomizations on our individual-level phylogeny and model testing in BioGeoBEARS on our species-level phylogenies, suggest no evidence for such time-clustered dispersal events. It has already been demonstrated that *Inga* communities across Amazônia were assembled through dispersal [25], which indicates that dispersal must have been an effective biogeographic force in this genus. *Inga* species are characteristic of rain forest environments, with very few found in tropical dry biomes and none in the savanna vegetation of central Brazil. We therefore suggest that *Inga* traversed the dry diagonal by dispersing through the dendritic net of humid gallery forests that mimic rain forests and which criss-cross central and northeast Brazil. The plausibility of this migration route is supported by the facts that some *Inga* species are characteristic of riparian habitats ([21]; figure 2) and that 21 *Inga* species with distributions primarily in either or both of Amazônia and Mata Atlântica also extend into the gallery forests of central Brazil, hence exemplifying the continuous populations required for dispersal through this dry diagonal region. A gallery forest route between Amazônia and the Mata Atlântica [18] seems a more likely explanation for *Inga* precisely because it is not time limited, despite being regarded as less important than the southern or northern routes by other authors (e.g. [7]). Whilst the gallery forests may have varied in extent across river basins because of varying historical rainfall patterns, we suggest that they have been constantly present as narrower or wider mesic ribbons facilitating continuous dispersal across a dry landscape. Our results point to the importance of the conservation of gallery forests to maintain rain forest habitat connectivity in the face of the massive conversion that the cerrado savannas of Brazil are suffering due to advancing industrial-scale agriculture.

*Inga’*s estimated crown age of 12.67 Ma [27], whilst pre-dating earlier estimates [24,26], still significantly postdates the drying of climate that started in the late Eocene/early Oligocene, intensified through the Miocene, and led to the establishment of the dry diagonal. Therefore, any scenario of ancient vicariance of a widespread rain forest formation seems unlikely to explain disjunct distributions of *Inga* in Amazônia and the Mata Atlântica. The dry diagonal comprises three distinct biomes, the tropical dry forests of the caatinga, the fire-prone savannas of the cerrado and the woodlands of the chaco. The caatinga was established as early as the Eocene-Oligocene [48] and whilst little is known of the geological age of the chaco, the lack of distribution points for *Inga* in both the north (caatinga) and especially south (chaco) of the dry diagonal (figure 1) suggests both are effective barriers. The cerrado, as a fire-adapted biome, is more recent, with fire-adapted plant clades that characterize it younger than 10 Ma, and often 5 Ma or less in age [49]; however, most dispersal events in *Inga* across the dry diagonal postdate even 5 Ma (figure 5).

*Inga* species, which do not have adaptations to dry environments (see figure 2), have therefore had to traverse the dry biomes of central Brazil by dispersal. We find little evidence that time-restricted opportunities for migration explain the Amazônia—Mata Atlântica biogeography of *Inga*, either in the middle to late Miocene (16−5 Mya; SE-NW route) or the Pliocene/Pleistocene (5 Mya—recent; NE route); in contrast, dispersal events are observed across the whole time period spanned by the *Inga* phylogeny (electronic supplementary material, figures S2 and S3). Our BioGeoBEARS analyses point to a single rate model of dispersal for *Inga* across the dry diagonal and that there is no evidence of dispersal events clustered at times consistent with the availability of SE-NW or NE routes (electronic supplementary material, table S4). We show that the temporal distribution of dispersals between Amazônia and the Mata Atlântica does not differ from null expectations of a more or less steady rate in the individual-level phylogeny (figure 5). The null model of constant dispersal probability per unit time (per unit branch length in a phylogeny) predicts more dispersal events in recent time, because this is where most of the branch length occurs in the phylogeny. This effect is magnified in an individual-level phylogeny because of the greater number of recent branches due to sampling of multiple accessions within many species, and potentially also to over-extension of terminal branches by accumulation of single-base changes in concatenated analysis of hybrid capture DNA sequence data (see [33]). However, the overwhelming evidence from null simulations and our formal model comparisons does not suggest temporal variation in dispersal rates between Amazônia and the Mata Atlântica over *Inga*’s entire evolutionary history.

Our study is the first to test formally if migration events between Amazônia and the Mata Atlântica are clustered in time in a plant clade. In addition, in this *Inga* study we used dense taxonomic sampling including multiple accessions within species that improves the ability to detect recent dispersal (i.e. 2 Ma or less), and such intraspecific sampling is lacking in other studies (e.g. [15,50]). Despite difficulties in comparing with published studies because of the absence of such intraspecific sampling, and lack of clarity on whether stem or crown ages are reported for Amazônia/Mata Atlântica clades, the pattern of dispersal events scattered through time is consistent with other dated phylogenetic studies of plant clades with species found in both Amazônia and Mata Atlântica. A Web of Science literature search uncovered eight studies where Amazônia—Mata Atlântica disjunctions ranged from 35 to 0.5 Ma, showing no clustering through time (table 1). For example, Machado *et al*. [51] documented six dispersal events from Amazônia to Mata Atlântica in *Ficus* L. ranging from 20 to 2 Ma.

**Table 1.** Ages of Amazônia—Mata Atlântica disjunctions from published, dated phylogenetic studies of plant clades.

taxon and publication	ages of Mata Atlântica—Amazônia disjunctions (crown age—stem age^a^)
*Amphilophium* Kunth (Bignoniaceae) [15]	28.4−32.2; 6.0−15.0; 3.0−6.0
Vrieseinae (Bromeliaceae) [11]	7.9−10.0
*Ficus* (Moraceae) [51]	15.0−20.0; 14.0−20.0; 7.0−10.0; 6.0; 4.0−10.0; 4.0; 2.0
Gesneriaceae [52]	21.1−31.7; 18.0−23.0
*Philodendron* Schott (Araceae) [53]	5.3−6.0; 3.7−6.8
*Pradosia* Liais (Sapotaceae) [50]	34.4; 11.7; 3.9−4.2
*Stigmaphyllon* A. Juss. (Malpighiaceae) [54]	10.0−22.0; 6.0−19.0; 0.5−12.0
Protieae (Burseraceae) [14]	10.0; 7.0−9.0; 5.0

^a^ Stem age only for single accessions of one species.

Our analyses clearly show that Amazônia is the ancestral area for clades containing Mata Atlântica *Inga* species or radiations and for populations of species widespread across both the Amazon and Mata Atlântica. *Inga* has been characterized as an ‘Andean centred’ group *sensu* Gentry [55] because its area of highest species diversity is on the western Amazônian flanks of the Andes mountains. Areas of highest species diversity do not always correlate with ancestral areas for species-rich genera in Amazônia (e.g. *Guatteria* Ruiz & Pav. [56]) or elsewhere (e.g. *Quercus* L. [57]), but in this case our analyses clearly identify Amazônia as the likely ancestral area, reinforced by the immediate sister group of *Inga* being two Amazônian species of *Zygia* P. Browne (*Z. inundata* (Ducke) H.C. Lima and *Z. sabatieri* Barneby & J.W. Grimes [27,58]). We have inferred 16.0 (BioGeoBEARS: coalescent species tree) to 19.7 (corHMM: individual-level tree) dispersal events to the Mata Atlântica from Amazônia with only 1.3–2.2 in the reverse direction (electronic supplementary material, table S5; non-integers reflect the average across 100 stochastic mappings [47]). In one case, *Inga edulis*, the individual-level phylogeny shows two independent dispersals from Amazônia to the Mata Atlântica. *Inga edulis* is widely used by people as a food plant [21], so in this case it is possible that humans may have influenced its distribution, with multiple assisted introductions to this area. The other species we have sampled with distributions in both Amazônia and Mata Atlântica (*I. thibaudiana*, *I. affinis*, *I. laurina, I. marginata, I. nobilis, I. capitata* Desv.*, I. striata* Benth.*, I. flagelliformis* (Vell.) Mart.) are not used by people, so their disjunctions presumably reflect natural processes. However, precisely dating these within-species dispersal events to prove this with certainty would require methods such as demographic modelling to be carried out using genome-scale data and additional sampling.

It may seem contradictory that we find endemic species in both the Mata Atlântica and Amazônia in the face of relatively frequent historical dispersal along the gallery forests that connect them. However, we also find many *Inga* species are confined to localized areas within Amazônia, the Mata Atlântica and the rain forests of Central America and the Chocó [21]. We have previously speculated [25,59] that speciation in *Inga* is mostly peripatric and driven by the dispersal that Dexter *et al*. [25] show to be prevalent in Amazônian *Inga* species. We suggest that whilst regional dispersal is prevalent across the *Inga* phylogeny, it is infrequent enough in some cases to allow localized species radiations, which could be a rapid process driven by the need to adapt to novel herbivore communities that attack young *Inga* leaves and represent a strong selective force (see [60,61]).

Our inference of continuous dispersal through time from Amazônia to the Mata Atlântica, through the inhospitable dry diagonal, suggests that in this setting, a dispersal-based model of biogeography is more appropriate than an event-based approach that emphasizes specific, time-limited episodes of connectivity. While more studies will be required to determine if *Inga* is typical, Dexter *et al*. [25] showed that dispersal-driven biogeographic patterns for *Inga* in Amazônia were shared by other, unrelated genera of trees. Furthermore, the lack of congruent phylogenetic and phylogeographic patterns found for birds in the Neotropics [62] also suggest a biogeographic model for this region that emphasizes dispersal. We propose that dispersal is as important as specific geological or climatic events in the biogeography and diversification of tropical forest trees, but that ability to disperse will be mediated by the intrinsic ecological attributes of taxa [59,63–65]. In the case of *Inga,* it is a rain forest-adapted ecology that confines it to gallery forests in the dry diagonal, and we suggest that other clades of rain forest-confined trees will show similar biogeographic patterns.

## Data Availability

Raw sequencing reads for each Inga accession have been deposited at NCBI’s Short Read Archive under either of the BioProject codes PRJEB8722 and PRJNA683762. Accession numbers for individual samples are provided in Supplementary Table S1 [66].
